# Non‐equivalent Results from Different Anteversion Measurements Methods for the Evaluation of the Acetabular Cup Orientation in Total Hip Arthroplasty

**DOI:** 10.1111/os.12445

**Published:** 2019-04-01

**Authors:** Thom E Snijders, Tom P C Schlösser, Steven M van Gaalen, Rene M Castelein, Harry Weinans, Arthur de Gast

**Affiliations:** ^1^ Clinical Orthopedic Research Center – mN Diakonessenhuis Zeist Zeist the Netherlands; ^2^ Department of Orthopaedic Surgery Diakonessenhuis Utrecht the Netherlands; ^3^ Department of Orthopaedic Surgery University Medical Center Utrecht Utrecht the Netherlands; ^4^ Department of Biomechanical Engineering TU Delft Delft the Netherlands

**Keywords:** Acetabular cup, Anteversion, Orientation, Total hip arthroplasty

## Abstract

**Objective:**

To determine the comparability among 10 radiographic anteversion methods for acetabular cup orientation in total hip arthroplasty (THA) found in the literature and the **“**gold” standard of assessing the anteversion with CT.

**Methods:**

This is a retrospective study that blindly compares 10 different conventional radiographic anteversion measurements with the “gold” standard, the measurement of anteversion on the transverse plane of the 3‐D images made with CT. The patient archiving and communications system (PACS) was systematically searched for subjects that had undergone a CT angiogram of the abdomen and lower extremities, including the pelvis, had at least one THA *in situ* and had undergone anterior‐posterior (AP) and cross‐lateral pelvic radiography between January 2013 and August 2016 in the Diakonessenhuis Hospital Utrecht/Zeist, a non‐academic institution. CT scans of patients (*n* = 16) were systematically collected. Three observers independently measured cup anteversion from radiographs, using a total of 10 different methods, and measured the “gold” standard on CT images. The outcomes of the 10 radiographic anteversion were compared in terms of linear correlation with the “gold” standard on CT images.

**Results:**

The correlations of the radiographic measured anteversions with the “gold” standard measured on CT images were 0.528 for the method of Liaw, 0.556 for Wan, 0.562 for the cross‐lateral method, 0.586 for Hassan, 0.594 for Dorr, 0.602 for Lewinnek, 0.624 for Widmer, 0.671 for the lateral CT, 0.747 for Ackland, and 0.771 for the method of Riten Pradham.

**Conclusion:**

Anteversion measurement methods represent different projectional angles of the acetabular cup in different planes around different axes. Therefore, they differ from the “gold” standard and are not interchangeable, as is shown by this study. We consider the anatomical anteversion in the transverse plane rotating around the longitudinal axis as the “gold” standard and recommend avoiding using the term anteversion for other projectional angles in different planes.

## Introduction

Acetabular cup orientation in total hip arthroplasty (THA) is considered of utmost importance to prevent aggravated wear, limited range of motion, and dislocation^1^, ^2^, ^3^, ^4^, ^5^, ^6^, ^7^. Over the past four decades, not much progress has been made with respect to optimal acetabular cup orientation, as demonstrated by the constant percentage of long‐term THA dislocations in large cohorts^8^, ^9^. Recent systematic reviews indicated that there is still no consensus on optimal acetabular cup orientation, because of mixed terminology and different projectional planes, used with several imaging modalities and different analysis methods^10^, ^11^.

**Figure 1:**
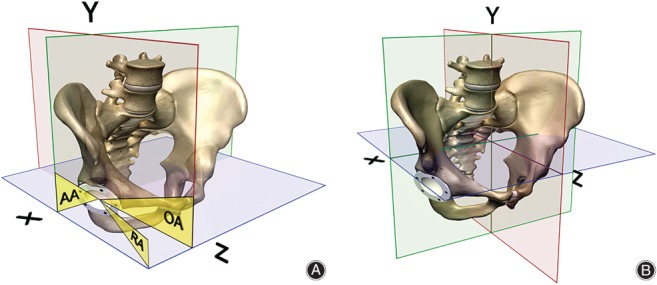
(A) Different spatial anteversion angles, defined by Murray, rotating around different axes^11^, ^15^. The colored planes show the three anatomical planes. The yellow angels describe the definitions in relation to the three anatomical planes: AA, anatomical anteversion in the transverse plane; OA, operative anteversion in the sagittal plane; RA, radiographic anteversion in a projectional plane. (B) Anatomical planes: green is coronal plane, red is sagittal plane, and blue is transverse plane. X is the transverse axis, Y is the longitudinal axis, and Z is the sagittal axis.

The orientation of the acetabular cup is historically evaluated using two angles: inclination and anteversion. Besides distinct terminology, such as abduction, tilt, flexion or lateral opening, several different definitions exist for inclination and, in particular, for anteversion^10^, ^11^. “Inclination” is mostly measured on anterior–posterior (AP) pelvic radiographs or on coronal plane projections of 3‐D imaging modalities and is an angle measured on a coronal plane that rotates around the sagittal axis. Because “anteversion” has been measured on lateral as well as cross‐lateral radiographs and on transverse plane projections of CT images, one has to realize that these different definitions are spatial varying angles measured on varying planes around different axes. First, anteversion measured on lateral radiographs is an angle on the sagittal plane around the transverse axis. Second, the cross‐lateral radiograph is measured on a plane in between the sagittal and transverse plane around an axis perpendicular to this plane. Third, the anteversion calculated with several varying algorithms from the ellipse of the acetabular cup projection on an AP pelvic radiograph is also a rotation measured on a plane, which is in between the transverse and sagittal plane with its corresponding perpendicular axis. Finally, anteversion measured on the transverse plane of a CT scan is rotating around the longitudinal axis. These different spatial angles were first described by Murray (Fig. 1A)^2^, ^12^, ^13^, ^14^, ^15^. The use of various “anteversion” angle definitions that are measured on different projectional planes has not led to comparable results. In our opinion, the 3‐D orientation of the acetabular cup, in reference to the anatomical planes, should be considered as the “gold” standard, because this is the anatomical anteversion measured on the transverse plane as described by Murray (Fig. 1B)^15^.

Optimal acetabular cup orientation recommendations should also be reproducible and usable in the preoperative planning, during surgery, and for postoperative evaluation. Therefore, to evaluate cup orientation properly, the definitions should be reproducible and consistent: preferably identical or at least comparable. However, it remains unclear whether the different conventional measurement methods described in the literature are comparable to the “gold” CT‐derived standard. The aim of this study is to evaluate the extent to which the different anteversion measurement methods described in the literature represent the “gold” standard.

## Materials and Methods

### *Study Inclusion and Exclusion Criteria*

After approval from the Institutional Review Board, the patient archiving and communications system (PACS) of the Diakonessenhuis Hospital Utrecht/Zeist, a non‐academic institution, was systematically searched for eligible subjects. The subjects were included if: (i) patients underwent a CT angiogram of the abdomen and lower extremities including the pelvis and had at least one THA *in situ*; (ii) they had undergone an AP and cross‐lateral pelvic radiography that enables the measurement of the different radiographic anteversion methods; and (iii) patients were only included if the imaging was done between January 2013 and August 2016. Exclusion criteria were: (i) previous ipsilateral hip surgery other than primary THA; (ii) malignant disease localized in the pelvis or femur; (iii) image series that were incomplete or with substantial contrast artifacts in the region of interest; and (iv) radiographs and CT scans that were obtained more than 3 months apart from each other.

### *Study Type*

This is a retrospective study that blindly compares 10 different conventional radiographic anteversion measurements with the “gold” standard, the measurement of anteversion on the transverse plane of the 3‐D images made with CT.

### *Study Procedure*

During the study period, following the standard protocol, angiographic CT scans were acquired in supine position using a 16‐channel multidetector CT system (Siemens Healthcare, Erlangen, Germany; slice thickness 0.5 mm) and intravenous contrast. Following protocol, AP‐pelvic radiographs were also taken in the supine position. The cross‐lateral pelvic radiograph was carried out in the supine position but with the contralateral hip flexed in 45° and placed on a small stand to keep the position stable. The direction of the radiation beam was parallel to the examination table, 45° to the long axis of the body, and the X‐ray film was opposite to the radiation beam^16^. There were no lateral pelvic radiographs available. Demographic characteristics were collected.

### *Anteversion Measurement Methods*

**Figure 2:**
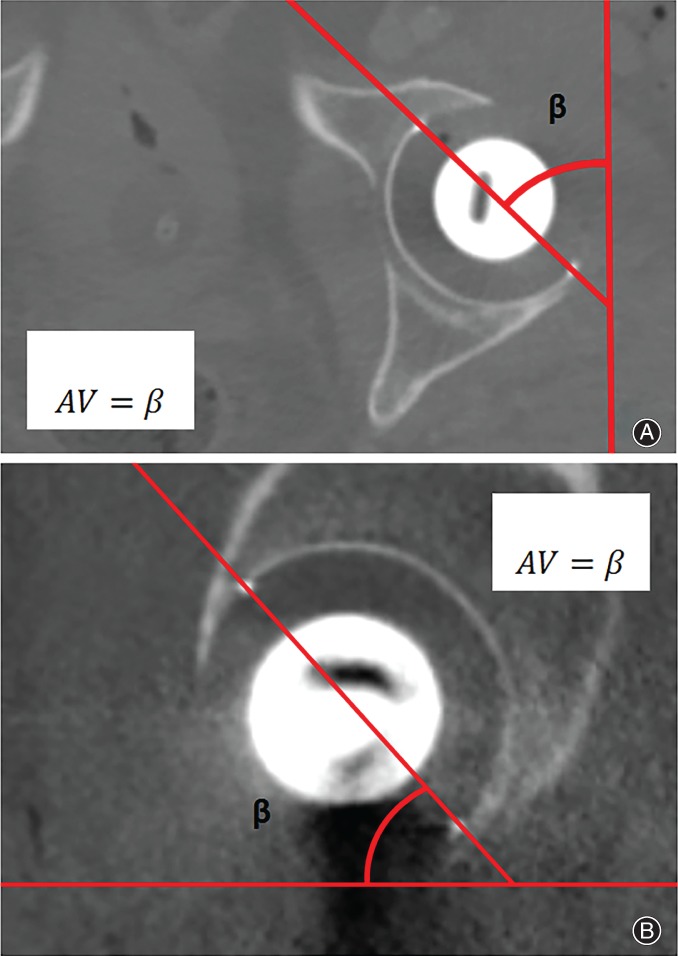
Category 1 methods. Definitions and algorithms of the included anteversion (AV) measurements methods with CT. The angle is measured by the opening of the cup in relation to the axis of the respective plane. (A) Transverse‐CT anteversion. (B) Sagittal‐CT anteversion.

**Figure 3:**
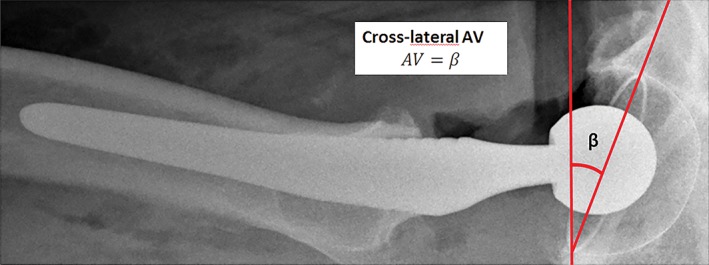
Category 2 method. Definition and algorithm of the included anteversion measurements on a cross‐lateral radiograph^16^. The angle is measured by the opening of the cup in relation to the axis of the respective plane. AV, anteversion; β, angle.

**Figure 4:**
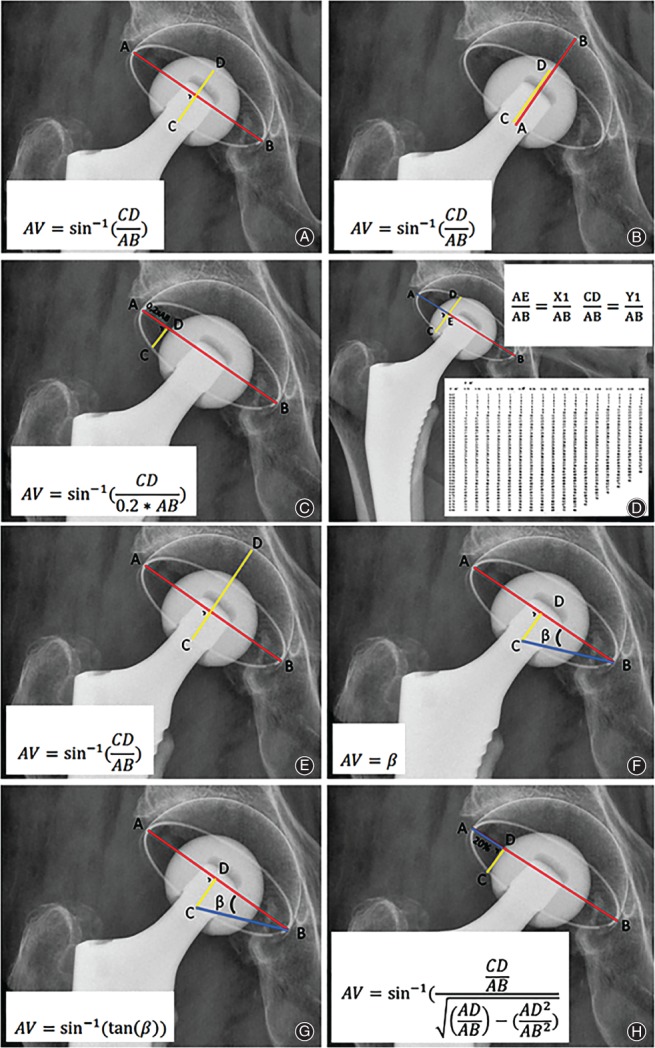
Category 3 methods. Definitions and algorithms of the included anteversion (AV) measurements methods with anterior–posterior radiographs. The respective distances are measured and filled in the respective formulas: (A) Lewinnek *et al.* 1, (B) Widmer *et al.* 7, (C) Riten Pradham^17^, (D) Ackland *et al.* 18, (E) Dorr *et al.* 19, (F) Wan *et al.* 20, (G) Liaw *et al.* 21, and (H) Hassan *et al.* 3 . β = angle.

All non‐automated methods for measurement of anteversion as found in two recent systematic reviews were included in this study^10^, ^11^. Studies comparing different anteversion measurement methods were also screened for additional measurement methods. A total of six measurement methods were identified from the systematic reviews^1^, ^2^, ^7^, ^14^, ^16^, ^17^, ^18^, ^19^. Three anteversion measurement methods were from other related articles^3^, ^20^, ^21^. The method of McLaren *et al.*, however, was excluded because of a non‐reproducible description of the measurement method used^22^. The method described by McCollum *et al.* performed anteversion measurement on lateral radiographs^14^. In our study, we used sagittal CT images for this method (Fig. 2). In total, 10 manual anteversion measurement methods were included and categorized with respect to the type of plane used for the measurement. Category 1 comprises methods using the anatomical planes, including the “gold” standard and the method of McCollum *et al.* (Figs 1A and 2)^14^, ^15^. Category 2 involves the cross‐lateral radiograph (Fig. 3)^16^. The third category includes methods that measure anteversion based on the ratios of the ellipse on an AP pelvic radiograph. These methods try to establish the radiographic anteversion by using different algorithms (Figs 1A and 4)^1^, ^3^, ^7^, ^17^, ^18^, ^19^, ^20^, ^21^. The radiographic anteversion is the angle measured on a spatial plane perpendicular to the acetabular cup axis.

Three observers were instructed in using the precise definitions and algorithms of the 11 different measurement methods. For intraobserver reliability, one observer measured the anteversion using the different methods in random order on three separate occasions, with a 2‐week interval. For interobserver reliability, all three observers performed the measurements on AP pelvic radiographs, and cross‐lateral pelvic radiographs using Rogan View Pro‐X (Rogan View Pro‐X, version 4.0.8.9, Rogan‐Delft B.V., Delft, the Netherlands). Finally, the anteversion method of McCollum *et al.* was measured on the sagittal plane and the anatomical anteversion was measured on the transverse plane of the CT scans of the pelvis, using HOROS Medical Image Viewer (Horos v2.0.2, Horos project, Annapolis, USA)^14^.

### *Statistical Analysis*

Statistical analyses were performed using IBM‐SPSS Statistics 23 (SPSS, Chicago, Illinois, USA). Continuous parameters were assessed and presented as mean ± standard deviation (range). Box plots were used to identify any outliers. For intraobserver and interobserver reliability, measured angles were compared within and between the observers using the intraclass correlation coefficient (ICC), with a one‐way random effects model for intraobserver reliability and a two‐way mixed effects model with absolute agreement for interobserver reliability. Validity of the different measurement methods was defined as compared to the anatomical anteversion of the acetabular cup on the transverse CT images that we consider to be the “gold” standard. The Pearson correlation coefficient was used for correlation analysis. We considered an alternative method that showed a correlation coefficient >0.80, with the “gold” standard as a good quality method that can be tolerated clinically. The outcomes of the different methods were also tested for differences of the mean using paired student *t*‐tests. The level of statistical significance was set at 0.05.

## Results

### *Population*

Sixteen THA on CT scans of 16 patients met the inclusion criteria. The primary THA were implanted between 2002 and 2016. Demographics are shown in Table 1. All CT angiograms were requested by a local vascular surgeon. All patients had a highly‐cross‐linked polyethylene uncemented monoblock acetabular cup (RM Pressfit cup, Mathys Ltd. Bettlach, Switzerland).

**Table 1:** Demographics

Parameters	Data (n = 16)
Number of females	11
Age (years)	75.9 ± 7.8 (62–88)* ^,^ †
Number of left sided total hip arthroplasty	7 (44%)
Uncemented acetabular component	16 (100%)
Monoblock	16 (100%)
Median cup size in mm	54 (50–60)†

* Mean and standard deviation (SD)

† Range.

### *Anteversion Measurement Results*

Measured anteversion data was normally distributed and box plots showed that there were no outliers. The anteversion measurement methods of Riten Pradham *et al.* (Fig. 4C) and Ackland *et al.* (Fig. 4D) were unable to calculate “anteversion” for two patients, who demonstrated relative high anteversion for the other measurement methods^17^, ^18^. Absolute outcomes of the different anteversion measurement methods are shown in Table 2. All methods showed excellent intraobserver and interobserver reliability: intraclass correlation coefficients for intraobserver and interobserver reliability varied between 0.921 and 0.997, and 0.871 and 0.996, respectively (Table 3).

**Table 2:** Different measurement methods outcomes

Anteversion measurement method	Category	n	Mean ± SD
Transverse CT^2^	1	16	26.6° ± 12.6°
Lateral CT^14^	1	16	25.2° ± 12.7°
Cross‐Lateral^16^	2	16	27.1° ± 11.7°
Lewinnek *et al.* 1	3	16	20.4° ± 10.4°
Widmer *et al.* 7	3	16	32.4° ± 13.0°
Riten Pradham *et al.* 17	3	14	37.0° ± 20.7°
Ackland *et al.* 18	3	14	16.6° ± 7.9°
Dorr *et al.* 19	3	16	38.8° ± 7.3°
Wan *et al.* 20	3	16	18.5° ± 8.4°
Liaw *et al.* 21	3	16	20.3° ± 10.5°
Hassan *et al.* 3	3	16	19.3° ± 10.4°

Outcomes of the different measurement methods and category are shown as mean and standard deviation (SD).

**Table 3:** Intraobserver and interobserver reliability analyses

Anteversion measurement method	Intraobserver reliability	Interobserver reliability	Absolute agreement (*P*‐value)	Correlation (*r*)
Transverse CT^2^	0.988 (0.973–0.995)	0.871 (0.736–0.948)	–	1
Lateral CT^14^	0.972 (0.938–0.989)	0.993 (0.983–0.997)	0.616	0.671
Cross‐Lateral^16^	0.991 (0.980–0.997)	0.984 (0.965–0.994)	0.847	0.562
Lewinnek *et al.* 1	0.997 (0.994–0.999)	0.996 (0.990–0.998)	0.032*	0.602
Widmer^7^	0.971 (0.935–0.996)	0.996 (0.991–0.999)	0.054	0.624
Riten Pradham^17^	0.978 (0.946–0.992)	0.988 (0.970–0.996)	0.009*	0.771
Ackland *et al.* 18	0.992 (0.981–0.997)	0.992 (0.980–0.997)	0.002*	0.747
Dorr *et al.* 19	0.995 (0.988–0.998)	0.990 (0.976–0.996)	0.000*	0.594
Wan *et al.* 20	0.947 (0.884–0.979)	0.950 (0.890–0.980)	0.008*	0.556
Liaw *et al.* 21	0.921 (0.831–0.969)	0.940 (0.869–0.977)	0.045*	0.528
Hassan *et al.* 3	0.980 (0.956–0.992)	0.959 (0.910–0.984)	0.016*	0.586

For intraobserver reliability analyses, differences between anteversion measurements were evaluated between multiple measurements of one observer using the ICC. For interobserver reliability analyses, differences between measured angles were evaluated between multiple measurements of three different observers using the ICC. Results of the linear correlation (Pearson correlation coefficient) were evaluated between the different anteversion measurement methods and the acetabular cup orientation on transverse CT. ICC is shown including the 95% confidence interval.

AV, anteversion; ICC, intraclass correlation coefficient.

* Significant (*P* = 0.05).

### *Differences of the Mean Outcomes and Linear Correlation Analysis*

**Figure 5:**
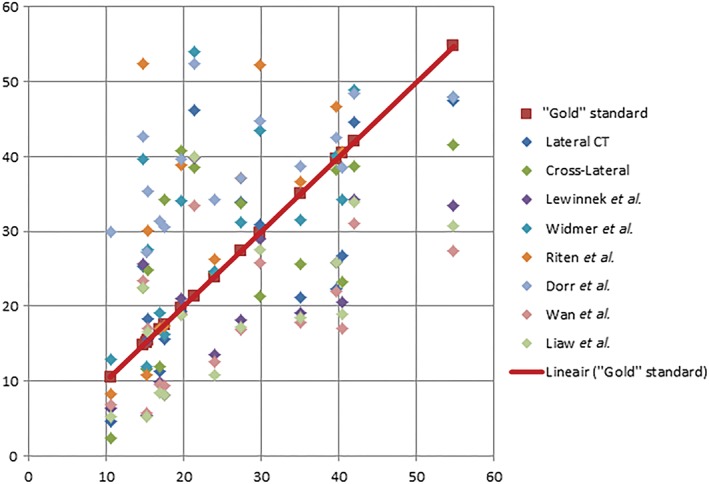
Results per patients for the different anteversion measurement methods on the x‐axis versus the “gold” standard on the y‐axis.

Three measurement methods (anteversion measured on the sagittal plane with CT, a cross‐lateral pelvic radiograph and the method of Widmer *et al.*) showed no significant difference in mean outcome as compared to our “gold” standard, the anteversion measured on transverse CT scans^7^. The other methods (all on AP‐pelvic radiographs) differed significantly from the cup orientation on transverse CT scans (Table 3). Correlation analyses revealed significant linear correlations varying between 0.528 and 0.771 for all methods when compared to the transverse version on CT scans (Table 3) (Fig. 5).

## Discussion

Multiple definitions for acetabular cup anteversion in THA exist. In order to study the relevance of acetabular cup orientation in relation to clinical outcome, it is of major importance that the measured orientation of different studies are comparable and lead to equivalent clinical guidelines for optimal acetabular cup placement. Therefore, this study compared different anteversion measurement methods with the “gold” standard method. In summary, although outcomes of three conventional measurement methods were on average the same as our “gold” standard, individual differences were wide. For this reason, the outcomes are neither directly comparable nor interchangeable (Table 3). This is the first study comparing all non‐automated measurement methods for acetabular cup anteversion with a “gold” standard and it provides an explanation as to why there is still no consensus on optimal acetabular cup orientation to date^10^, ^11^.

Our study demonstrates that none of the included methods can function as a substitute for the “gold” standard as they all do not reach the threshold for correlation analyses. Studies investigating so‐called “safe zones” for acetabular cup orientation provide recommendations that cannot be applied to other definitions without discrepancies. For example, using cross‐lateral radiograph based recommendations as a target during surgery, while changing the anteversion following the operative anteversion definition of Murray will not give the expected result, because it rotates around another axis (Fig. 1A)^15^. Our results did show that a cross‐lateral radiograph, a lateral radiograph, and an AP radiograph were not statistically significantly different from the “gold” standard. This might suggest that these methods could be used as a surrogate. However, it is more likely that this finding is caused by the small number of patients. With a larger study group this effect would probably also be statistically significantly different, because the measurements concerned use different spatial angles.

The differences in these methods lies in the direction of their axis where the angle rotates around. For the “gold” standard the axis of rotation is the longitudinal axis (Y in Fig. 1B). Surrogate measurement methods rotate around different axes. The method of McCollum *et al.* rotates around the transverse axis (X in Fig. 1B), while the category 3 methods rotate around an axis perpendicular to a plane between the transverse and sagittal plane^14^. Thus, it rotates around an axis somewhere between the longitudinal (X) and the transverse axis (Y). This specific axis is dependent on the orientation of the acetabular cup. For an example, one could have two patients with both an anteversion of 30° with the method of Widmer *et al.* and have inclinations of 15° and 60°, respectively^7^. If one uses the “gold” standard in both patients, differences in anteversion will be measured. The patient with an inclination of 60° will have a relatively low anteversion measured with the “gold” standard, while the patient with an inclination of 15° will have a relative high anteversion with the “gold” standard. Thus, compared to the “gold” standard the methods using an ellipse have a relationship with the inclination. Another factor involving the category 3 methods is that it is impossible to define if the acetabular cup has anteversion or retroversion with all methods that use the ellipse on an AP pelvic radiograph (Fig. 4).

### *Limitations*

Several other factors could cause diverging measurements and are limitations to our study: measuring error, position of the patient, orientation of the pelvis, position of the radiation beam of the radiograph, and intervariability of the anatomy of the individual patient. The measuring error proved to be small, as shown by the excellent intraobserver and interobserver reliability of all methods (Table 3). Patient positioning may have influenced our results, despite the similar patient positioning for different imaging modalities and that it was defined in protocols. Still, slight deviations cannot be excluded. Standardized orientation of the pelvis is more difficult. For instance, the study of Lewinnek *et al.* did standardize the pelvic tilt by adjusting the anterior pelvic plane until it was parallel to the table^1^. Most other studies and our study did not carry out this adjustment. Patient positioning and adjusting the pelvis so that it is parallel to the table can be changed before measuring the acetabular orientation. This is in contrast to the patients’ anatomy, which is fixed. The patients’ anatomy determines the reference plane from where the angles are measured.

This study had some other limitations. First, there were some missing values. Using the methods described by Riten Pradham *et al.* and Ackland *et al.*, we could not calculate the anteversion for two cases, because these two had relatively increased anteversion^17^, ^18^. This may have introduced a selection bias, which could affect the results. In contrast, this shows that these methods are not suitable for clinical use in a wide range of cup orientations. The second limitation is the relatively small sample size, which introduces a risk for a type 2 error. In our database, there were no more THA patients available with CT angiogram images, cross‐lateral‐pelvic radiographs and AP‐pelvic radiographs acquired in the same position. Because of the heterogeneity of our study population, we believe our results generally hold true. However, we do realize that a larger cohort would have given the article more statistical validity. A third limitation is the “gold” standard itself. To our knowledge, there is no study that has validated the “gold” standard. Fourth, with a change in pelvic rotation, tilt or obliquity, a different anteversion is measured. For example, there could be a small change in orientation of the pelvis of the patient in the supine position between the radiographic imaging table and the CT imaging table^23^. Fifth, there might be an increased measuring error with the cross‐lateral pelvic radiograph, because the pelvis could tilt posteriorly. This occurs particularly in patients with contralateral osteoarthritis of the hip with a flexion contracture. Finally, including the methods based on a software program that defines the anteversion on an AP‐pelvic radiograph would have made this study complete. Unfortunately, these resources were not available. Nevertheless, these methods are based on the ellipse as well and are also subject to the influence of the inclination and possible retroversion of the acetabular cup, as described above.

### *Conclusions*

This study shows that there is no correlating surrogate anteversion measurement method to substitute the “gold” standard, anteversion measured in the transverse plane around the longitudinal axis on a CT scan. Consequently, studies evaluating acetabular cup orientation with different methods are difficult to standardize and cannot be compared. Therefore, it is difficult to provide a recommendation concerning the optimal acetabular cup orientation^10^, ^11^. We consider the anatomical anteversion in the transverse plane rotating around the longitudinal axis to be the “gold” standard and recommend avoiding using the term anteversion for other projectional angles in different planes.
